# Impact of medical comorbidities on mental disorder pathoplasticity in elderly patients: a case report

**DOI:** 10.1192/j.eurpsy.2025.1742

**Published:** 2025-08-26

**Authors:** F. Mayor Sanabria, M. Fernández Fariña, M. D. P. Paz Otero, E. Expósito Durán, C. Regueiro Martín-Albo, J. V. De Leon Quiroz, M. I. Ramos García

**Affiliations:** 1 Instituto de Psiquiatría y Salud Mental, Hospital Clínico San Carlos, Madrid; 2 Servicio de Geriatría, Hospital San Pedro de Alcantara, Cáceres, Spain

## Abstract

**Introduction:**

Careful qualitative analysis of medical comorbidities in psychogeriatric patients is frequently overlooked in clinical practice, although these individuals often present with complex clinical pictures where multiple age-related somatic conditions can influence or alter the psycopathological presentation of mental disorders. This interplay can significantly affect both diagnosis and management, complicating the therapeutic approach and influencing prognosis.

We present the case of an 81-year-old male with a history of schizoaffective disorder admitted to our Psychiatry Unit due to an episode of marked motor inhibition and delusions. This case is notable for its complex clinical presentation, requiring a broad differential diagnosis, and serves as a representative example of the challenges in managing psychogeriatric patients with overlapping psychiatric and neurological comorbidities.

**Objectives:**

1. To describe the clinical particularities of this case, focusing on relevant psychogeriatric comorbidities and related changes in pathoplasticity.
2. To review the available evidence regarding the characteristics and management of comorbid neurological disorders in psychogeriatric patients.

**Methods:**

The patient’s clinical history was reviewed, including complementary tests such as brain MRI and PET-CT scans. Additionaly, a literature search was carried out, focusing on psychiatric and neurological comorbidities in elderly patients with a history of psychotic and affective disorders.

**Results:**

The patient’s clinical course reveals a significant change in the presentation of symptoms starting at the age of 70. Prior to this age, episodes were characterized mainly by inhibition, mutism, and delusional guilt. However, from the age of 70, there is a notable shift to more complex presentations with both manic and psychotic symptoms, including persecutory delusions, hyperactivity, and religious ideation, alongside periods of mixed affective states with significant affective lability. The development of probable drug-induced parkinsonism, confirmed by a negative DaTSCAN, and neuroimaging findings suggestive of a neurodegenerative process akin to Alzheimer’s disease further complicated the clinical picture. Therapeutic interventions included psychopharmacological adjustments and electroconvulsive therapy, resulting in partial stabilization.

**Conclusions:**

1. Managing psychogeriatric patients requires addressing comorbidities with a flexible, symptom-based approach.
2. Neurodegenerative processes can alter prognosis by increasing relapse risk and changing symptom patterns.
3. A multidisciplinary approach is crucial for optimizing care in complex psychogeriatric cases.

**Disclosure of Interest:**

None Declared

